# Supplementary material for: Polymorphisms in Pfkelch13 domains before and after the introduction of artemisinin-based combination therapy in Southwest Nigeria
Source: PLoS One. 2025 Mar 31;20(3):e0316479. doi: 10.1371/journal.pone.0316479 (PMC11957316; doi:10.1371/journal.pone.0316479)

Samples: 13071
Bases: 1107
Average spacing: 12

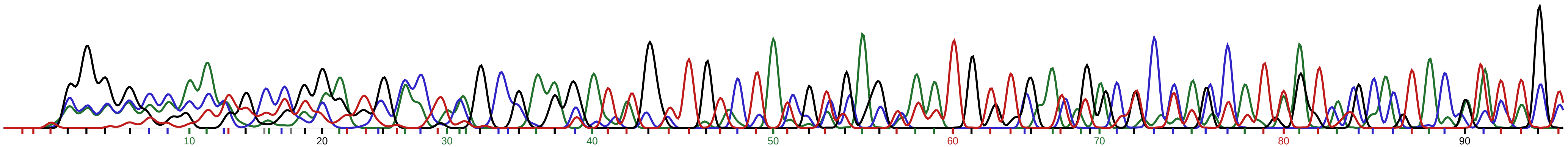

T T T G G G G C C A A C T G - A C - G G A T T C G T A C T A A A G C C G A G C A T T G T T G T C T A T G T G A G T A A T G T T C G A T A G A C G C C A C C A T T A T A C C C T A C G C T T G T

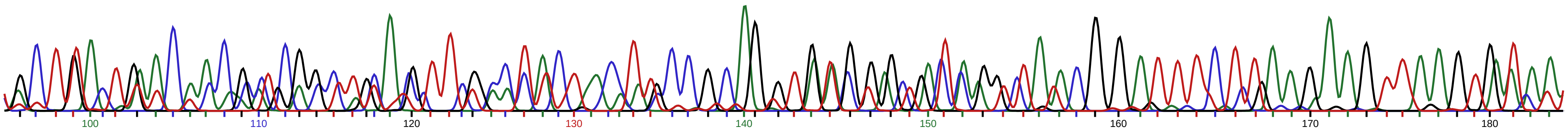

G C T T A C T G A C A A C G C T C G G T T G C A G T T C G A C T A C T A C A T T C C G C A G G T A T G G G T A T A G T T A A C G G A T T T C T T A A G A A G T T A A G T G T A A

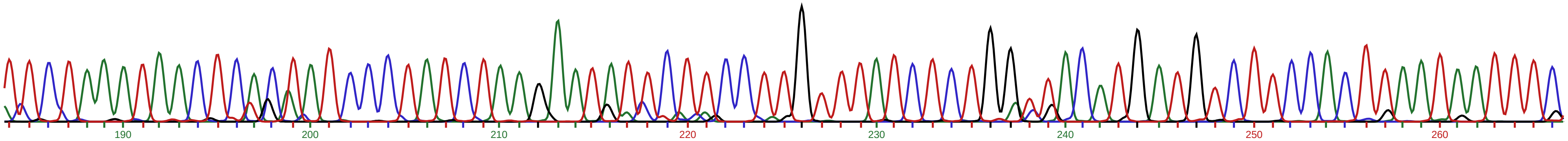

T T C T A A A T A A C T C A C T A T C C C T A T C T A A G A A T A T T C T T C C T T G T T T A T C T C T G G T T A C A T G A T G T C T T C C A C T T A A T A A T T T C

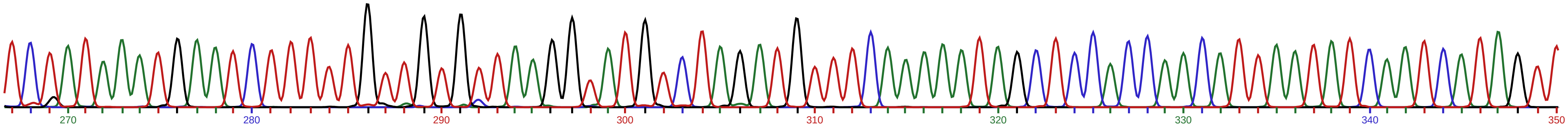

T C T A T A A A T G A A T C T T T T T G T T G T G T T A A G G T A T G T C T A G A T G T T T C A A A A A T A G C T C C A C C A A C A T T A A T A T C A A T C A T A G T T

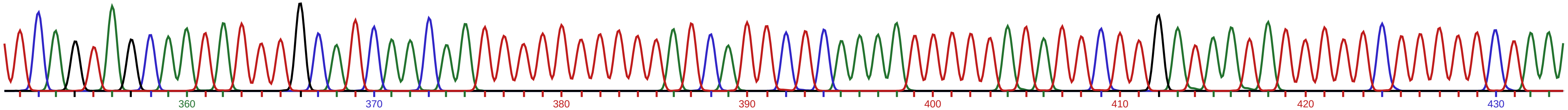

T C A G T A G C A A T A T T T G C A T C A A C A A T T T T T T T T T A T C A T T C T C A A A A T T T T T A T A T T C T T G A T A A T A T T T T T C T T T T T C T A A

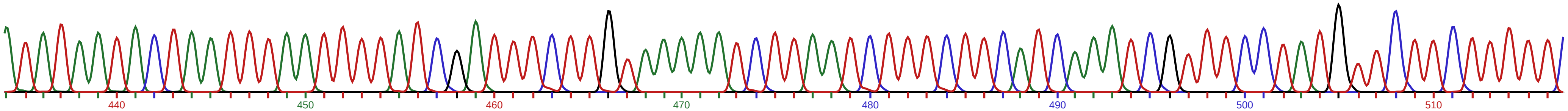

A T A T A A T A C T A A T T T A A T T T T A T C G A T T T C T T G T A A A A A T C T T A A T C T T T C T T C A T C A A A T C G T T T C C T A T G T T C T T C T T T T T

Samples: 13071
Bases: 1107
Average spacing: 12

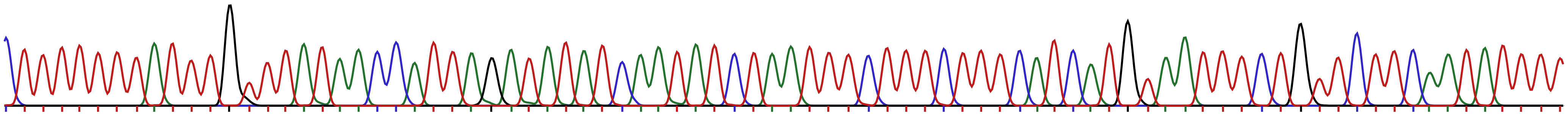

520 530 540 550 560 570 580 590 600
C T T T T T T T A T T T G T T T A T A A C C A T T A G A T A T A T C A A T A T C T A A T T T C T T T C T T T C A T C A T G T A A T T T C T G T T C T T C A A T A T T T T

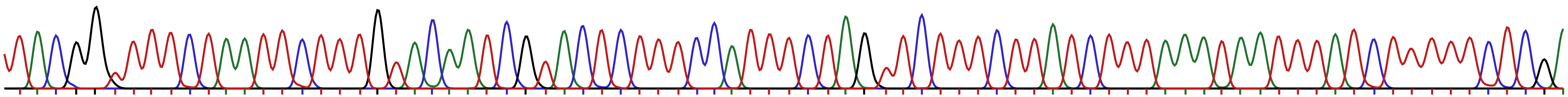

610 620 630 640 650 660 670 680
T A C G G T T T T C T A A T T C T T T G T A C A A T C G T A C T C T T T C C A T T T C T A G T T C T T T C T T A T C T T T A A A T A A T T T A T C T T T T T C T C G A

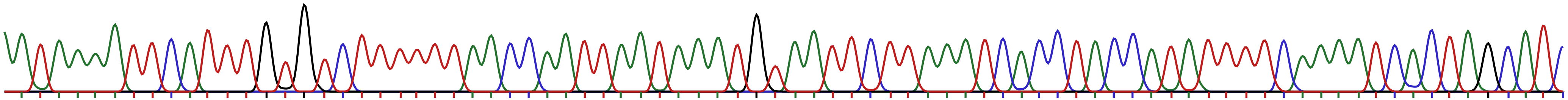

690 700 710 720 730 740 750 760
A T A A A A T T C A T T T G T G T C T T T T T T A A C C A A T T A A T A A A T G T A A T T C T T A A A T C A C C T A C C A T A T T T T C A A A A T C A C T A G C A T C

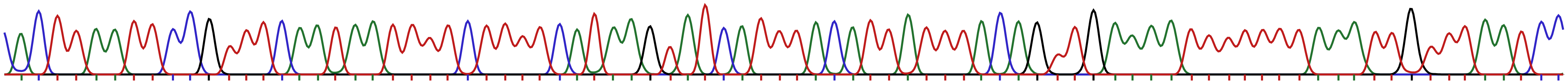

770 780 790 800 810 820 830 840 850
A C T T A A T T C C G T T T C A A T A A T T T T C T T T T C A T A A G T A T C A T T T A C A T T A T T T A C A G T T G A A A A T T T T T T T A A A T T G T T T A A T C C

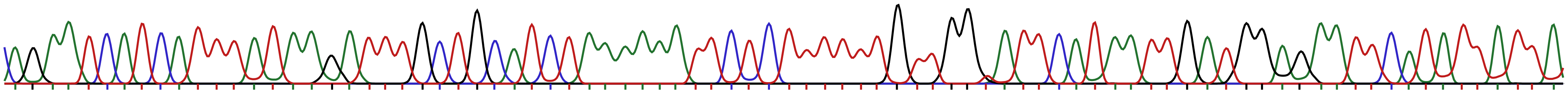

860 870 880 890 900 910 920 930
A G A A T C A T C A T T T A T A A G A T T T G C T G C A T C T A A A A A A T T C T C T T T T T T G T T G G T A T T C A T A A T T G A T G G A G A A T T C A T A T T A T T A

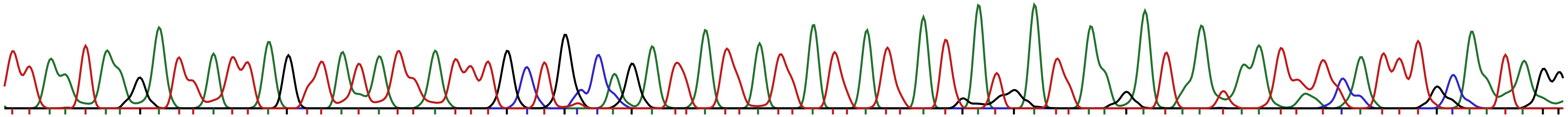

940 950 960 970 980 990 1000 1010 1020
T T A A T A A G A T T A T T A G T T A T A T T A T T T G C T G C C A G A T T A T T A T T A T T A T T A T T A T G A T G A T T A A G A T A A T A A T T T C A T T T G C A A T A G G

Samples: 13071
Bases: 1107
Average spacing: 12

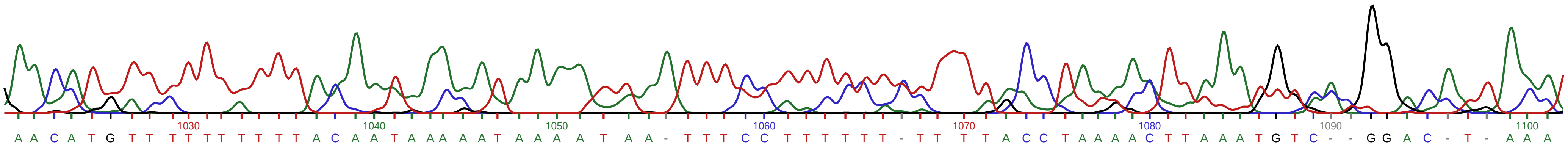

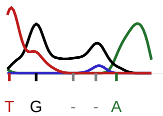

Supplement: Supporting information 2 — (ZIP) [file pone.0316479.s002.zip › 192KN2R_PREMIX_Plate_KELCH2_H07.pdf]
